# A High-Throughput Method as a Diagnostic Tool for HIV Detection in Patient-Specific Induced Pluripotent Stem Cells Generated by Different Reprogramming Methods

**DOI:** 10.1155/2019/2181437

**Published:** 2019-08-05

**Authors:** Daniela Hübscher, Sabine Rebs, Luis Haupt, Thomas Borchert, Celina Isabell Guessoum, Franziska Treu, Steffen Köhne, Andreas Maus, Mario Hambrecht, Samuel Sossalla, Ralf Dressel, Angela Uy, Mark Jakob, Gerd Hasenfuss, Katrin Streckfuss-Bömeke

**Affiliations:** ^1^Clinic for Cardiology and Pneumology, University Medical Center Goettingen, Goettingen, Germany; ^2^DZHK (German Center for Cardiovascular Research), Partner Site Goettingen, Goettingen, Germany; ^3^Department of Internal Medicine II, University Medical Center Regensburg, Regensburg, Germany; ^4^Institute of Cellular and Molecular Immunology, University Medical Center Goettingen, Goettingen, Germany; ^5^Institute for Medical Microbiology, University Medical Center Goettingen, Germany; ^6^Department of Otorhinolaryngology, Klinikum der Universität München, Ludwig-Maximilians-Universität München, Munich, Germany

## Abstract

Induced pluripotent stem cells (iPSCs) provide a unique opportunity for generation of patient-specific cells for use in translational purposes. We aimed to compare iPSCs generated by different reprogramming methods regarding their reprogramming efficiency, pluripotency capacity, and the possibility to use high-throughput PCR-based methods for detection of human pathogenic viruses. iPSCs from skin fibroblasts (FB), peripheral blood mononuclear cells (PBMCs), or mesenchymal stem cells (MSCs) were generated by using three different reprogramming systems including chromosomal integrating and nonintegrating methods. Reprogramming efficiencies were in accordance with the literature, indicating that the parental cell type and the reprogramming method play a major role for the reprogramming efficiencies (FB: STEMCCA: 1.30 ± 0.18, Sendai virus: 1.37 ± 0.01, and episomal plasmids: 0.04 ± 0.02; PBMCs: Sendai virus: 0.002 ± 0.001, episomal plasmids: 0) but result in the same characteristics of pluripotency. We found the highest reprogramming efficiencies for MSC with 3.32 ± 1.2 by using episomal plasmids. Since GMP standard working procedures and screening units need virus contamination-free cell lines, we studied HIV-1 contamination in the generated iPSCs. We used the high-throughput cobas® 6800/8800 system, which is normally used for detection of HIV-1 in plasma of patients, and found that footprint-free reprogramming methods as episomal plasmids and Sendai virus are useful for the described virus detection method. This fast, cost-effective, robust, and reliable assay demonstrates the feasibility to use high-throughput PCR-based methods for detection of human pathogenic viruses in ps-iPSC lines that were generated with nongenome integrating reprogramming methods.

## 1. Introduction

The ability to generate induced pluripotent stem cells (iPSCs) from somatic cells of patients offers a great opportunity to model human diseases and creates a powerful tool for drug screening and the development of new grafts for transplantation.

Until now, numerous methods have been developed to generate iPSCs. Compared to the original studies of Takahashi et al., these methods resulted in increased reprogramming efficiencies and furthermore in footprint-free iPSCs lacking viral sequence integration into their genomes [1, 2]. Reprogramming of adult human skin fibroblasts was first described in 2007 [2] with a reprogramming efficiency of ~0.02% 30 days after retroviral transduction of the reprogramming factors [2]. To minimize vector sequences that were integrated into the reprogrammed iPSC genome, a humanized version of a single cassette reprogramming vector became popular offering the possibility of Cre-Lox-mediated transgene excision. For this vector termed STEMCCA, reprogramming efficiencies of 0.1-1.5% are reported [3]. Furthermore, nonintegrating viruses for reprogramming were described in the literature. One of these is the Sendai virus that as an RNA virus does not enter the nucleus and is diluted out of the cells ~10 passages after transduction. Sendai virus was shown to be able to reprogram neonatal and adult human fibroblasts in about 25 days with a high efficiency of 1% [4]. Another integration-free and nonviral method for reprogramming is the use of episomal plasmids expressing transiently the reprogramming factors. Using the OriP/EBNA-based plasmids in combination with p53 suppression and nontransforming L-Myc, the reprogramming efficiency can be increased to 0.1% [5–7].

Patient-specific iPSCs offer the perspective of future use in transplantation studies; hence, iPSC biobanks were generated for allogeneic transplantation experiments. Some iPSC lines have already been used for several years, and frozen cells obtained many years ago can be found in several laboratories. It is known that human cells can harbor different human pathogens and resemble therefore a potential risk for the recipients of a transplant to become infected when virus screening is missing [8]. Usually, infected iPSC lines were generated from already virus-infected patient material [9]. Different human pathogenic viruses can be found in patient and donor materials like the human immunodeficiency virus type 1 (HIV-1) or hepatitis viruses as hepatitis B virus (HBV) and hepatitis C virus (HCV), and the risks depend on the risks of the studied patients. However, contaminations of cell cultures can also be introduced secondarily by laboratory personnel or from other infected cells when handled at the same time and place. Similar problems were shown for mycoplasma contaminations and cross contaminations of cell culture [10]. Working with human cell lines, especially iPSC lines, represents per se no increased risk, but an infection of these cells with human pathogens enhances the potential risk of the cell culture work. Until the infection status of the donor or the cells is not clearly determined, primary cells or cell cultures should be categorized as a safety risk group 2.

Previous studies have compared different reprogramming methods and differentiation capacities of iPSC lines, mostly from different donors, at different passages and with use of different culture conditions [11, 12]. To determine if the reprogramming method influences the use of high-throughput PCR-based methods for detection of human pathogenic viruses in patient-specific- (ps-) iPSC lines, we generated iPSC lines with three frequently used reprogramming methods: episomal vectors, lentivirus, and Sendai virus. Because the sex, age, and genetic background of the parental cells could influence the resulting pluripotency of the hiPSCs generated, we used the same fibroblast population for each reprogramming method. Furthermore, we have used standardized hiPSC growing conditions (Geltrex and chemically defined E8 conditioned media). In line with the literature, we show in this study that the parental cell type plays a major role for the reprogramming efficiency and that the reprogramming methods indeed result in different reprogramming efficiencies but the same characteristics of pluripotency. In our study, mesenchymal stem cells (MSCs) are the most promising somatic cell source with a reprogramming efficiency of 3.32 ± 1.2 by using episomal plasmids. Furthermore, we demonstrate for the first time the feasibility to use high-throughput PCR-based methods for detection of human pathogenic viruses in ps-iPSC lines that were generated from somatic cells with nongenome integrating reprogramming methods. Therefore, a fast and cost-efficient method could be used to screen routinely the *in vitro* cell culture for contamination.

## 2. Materials and Methods

### 2.1. Patients

Somatic cell donors presented here have been randomly selected based on their generation method (Table 1, Supplemental Table 3). STv-iPSC-FB3 (FB3), STv-iPSC-FB5 (Kera2-iPSC1), and STv-iPSC-42 (1-RBM20) were characterized and described in our previous studies and included in this study because of the STEMCCA virus reprogramming method [13, 14].

### 2.2. Somatic Cell Isolation and Cultivation

This study was approved by the Ethics Committee of the University Medical Center Göttingen (approval number: 21/1/11, 10/9/15, and 3/4/17) and carried out in accordance with the approved guidelines. Samples from donors were collected under signed informed consent prior to the participation in the study. The fibroblast culture was established from skin punch biopsies, MSC culture from tissue from the inferior nasal concha, and PBMC from blood samples of the donors. The skin punch biopsies (3.5-4 mm) were taken aseptically by a surgeon, placed in DMEM containing penicillin (100 U/mL)/streptomycin (100 *μ*g/mL), transferred at the soonest to the lab, and cut into pieces of 1–1.5 mm side length. The pieces were transferred in cell culture dishes with epidermis upside down and cultivated in fibroblast growth medium composed of DMEM supplemented with 10% FCS, 1% NEAA, glutamine (2 mmol/L), *β*-mercaptoethanol (50 *μ*mol/L), penicillin (50 U/mL)/streptomycin (50 *μ*g/mL), and bFGF (10 ng/mL) at 37°C with 5% CO_2_ atmosphere without moving the dishes. Isolation of MSCs was performed from tissue from the inferior nasal concha (<1 g), which was obtained from healthy individuals. Samples were transferred to 10 mL of a 0.9% NaCl solution after tissue resection, and MSCs were isolated according to an adapted version of the isolation protocol described earlier [15]. Cell cultivation was performed in MSC growth medium (94% DMEM (Thermo Fischer Scientific, Waltham, Massachusetts), 5% human platelet lysate (PL BioScience GmbH, Aachen, Germany), 1% penicillin/streptomycin (Thermo Fischer Scientific), 1% sodium pyruvate (Gibco Invitrogen, Karlsruhe, Germany), and 0.04% heparin (Biochrom, Berlin, Germany)) at 37°C and 5% CO_2_ as described in an earlier publication [16]. For isolation of peripheral blood mononuclear cells (PBMCs), stabilized blood samples were diluted 1 : 1 in PBS. 6 mL of the blood cells was carefully separated by Ficoll gradient centrifugation for 25 min at 400 g. The generated interphase was washed once with cold PBS. After centrifugation (600 g for 5 min at 4°C), the obtained cells were resuspended in blood medium (StemPro basal medium with StemPro34 supplement, 2 U/mL EPO, 100 ng/mL FLT-3, 100 ng/mL SCF, 20 ng/mL IL-3, and 20 ng/mL IL-6) and cultivated on 12-well plates (1–1.5 × 10^6^ cells/well).

Usually, transduction and transfection experiments were performed before passage 3 (p3). A daily medium change was carried out.

### 2.3. Generation and Culture of ps-iPSCs

For STEMCCA-based transduction, a total of 2 × 10^4^ fibroblasts were plated in one well of a 6-well tissue culture plate and transduced with lentiviral particles (STEMCCA cassette) as described before [13]. The STEMCCA cassette is a humanized excisable system containing all four reprogramming factors OCT4, SOX2, KLF4, and c-MYC in a single “stem cell cassette” (pHAGE2-EF1aFull-hOct4-F2A-hKlf4-IRES-hSox2-P2A-hcMyc-W-loxP). For Sendai-based transduction, the protocol was described earlier [17]. Briefly, 7.5 × 10^4^ fibroblasts or 3 × 10^5^ hPBMCs were seeded in 2 wells of a 12-well plate, respectively, 2 days before transduction. Sendai virus from the CytoTune™-iPS 2.0 Sendai Reprogramming Kit (Thermo Fisher Scientific) was used at a MOI of 5/5/3 (KOS/hc-myc/hKLF4) and added to the cells in fresh medium. Medium was changed every other day, and blood medium was supplemented with 500 *μ*mol/L sodium butyrate (Sigma-Aldrich). At day 7, cells were transferred to a Geltrex-coated (Thermo Fisher Scientific) 6-well plate and medium was changed to E8 medium (Thermo Fisher Scientific). For plasmid-based integration-free reprogramming, the protocol was described earlier [5]. Briefly, 4‐5 × 10^5^ cells were used for electroporation with the NHDF Nucleofector Kit (Lonza): cells were suspended in nucleofection solution, and 1-2 *μ*g of the plasmids pCXLE-hSK, pCXLE-hUL, and pCXLEhOct3/4-shp53-F were used per experiment. Electroporation was done with the Nucleofector II (Lonza) with the program P22 or U23. Cells were plated on 6-well plates in fibroblast medium with 5 *μ*mol/L prosurvival factor (Millipore) and 500 *μ*mol/L sodium butyrate. Medium was changed every other day with cell type-specific medium with 500 *μ*mol/L sodium butyrate. At day 7 post transfection, transfected cells were harvested and plated on a Geltrex-coated 6-well plate for picking iPS-like colonies. About 3-4 weeks after the specific reprogramming procedure, colonies with iPS-like morphology were picked mechanically and gave rise to the different stem cell lines. Newly established stem cell lines were passaged with Versene solution (Thermo Fisher Scientific) and cultivated in E8 medium five passages before being used for experiments.

### 2.4. Sample Preparation for HIV-PCR Testing

Fibroblasts or iPSCs were lysed as described in the SV Total RNA Isolation System (Promega). 1 mL lysate buffer was used for initial lysis of 1 × 10^6^ cells, filled up with lysis buffer to 3.5 mL, and transferred to the cobas® 6800/8800 system in combination with the cobas® HIV-1 test. HxV H (+) C (human plasma containing 0.001% synthetic Armored RNA of the HIV-1 group M with high titer packaged in envelope protein MS2) and HxV L (+) C (human plasma containing 0.001% Armored RNA of the HIV-1 group M with low titer packaged in envelope protein MS2) were used as positive controls for HIV-1. As an internal negative control, human plasma without HIV-1 detection was used. The sensitivity of the PCR assay was determined as about 20 copies per reaction (cp/mL).

## 3. Results

### 3.1. The Reprogramming Efficiency Is Dependent on the Used Method and the Parental Cell Source

**Figure 1:**
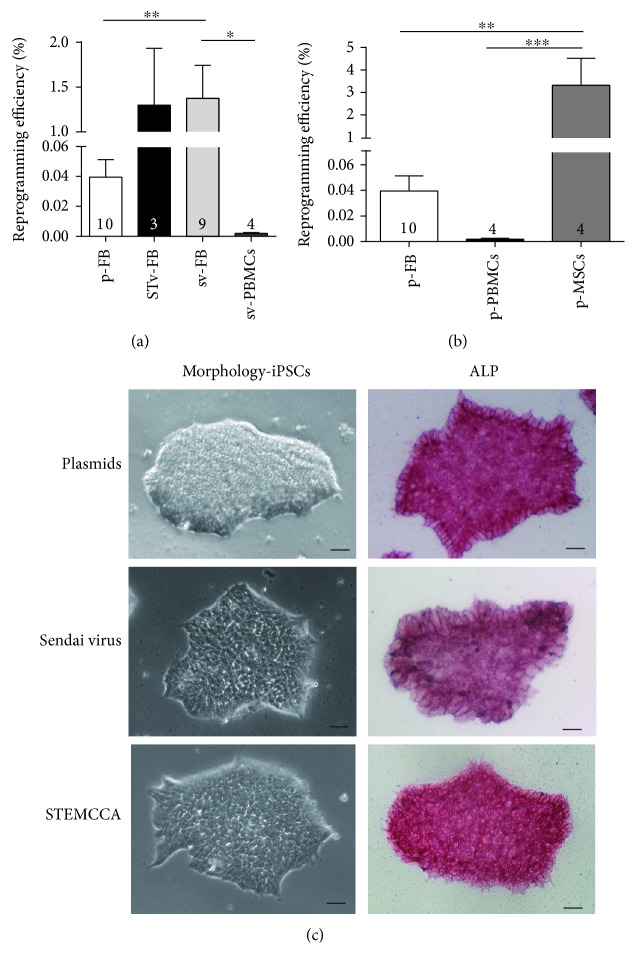
Generation and characteristics of hiPSCs derived from skin fibroblasts (FB). (a) Reprogramming efficiency for generation of PMBC-hiPSCs and FB-hiPSCs based on different reprogramming methods. Reprogramming efficiency was evaluated as total iPSC colonies obtained up to day 25 from 4.8 to 40 × 10^4^ starting FB (dependent on the used method); *n* = 10 (p-FB = plasmids), *n* = 3 (STv-FB = STEMCCA), and *n* = 9 (sv-FB = Sendai virus). 3 × 10^5^ PBMCs were used for reprogramming by sv; *n* = 4 (sv-PBMCs). ^∗^ *P* ≤ 0.05; ^∗∗^ *P* ≤ 0.01. *n* (numbers) are defined as reprogramming experiments of different somatic cell sources by the mentioned method. (b) Reprogramming efficiency for generation of FB-iPSCs (*n* = 10), PBMC-iPSCs (*n* = 4), and MSC-iPSCs using episomal plasmids. 5 × 10^5^ MSCs were used for reprogramming by plasmids; *n* = 4. (c) Morphology of iPSCs. iPSCs show human ES-like morphology and are positive for alkaline phosphatase (ALP). Scale bar: 100 *μ*m.

FB, PBMC, and MSC were reprogrammed by using three different systems: the STEMCCA virus system as a genome integrating method and episomal plasmids and the Sendai virus system as nonintegrating reprogramming methods. The numbers of ps-iPSC lines generated with three different systems were summarized in Table 2 and Supplemental Table 3. Our data of reprogramming FB and PBMCs showed that success rates (number of donors with successful generation of iPSCs) for FB are highest when the Sendai virus or the STEMCCA virus system was used (100%) compared to plasmids (89%) (Supplemental Table 3). Reprogramming of PBMCs was most successful by using Sendai virus for reprogramming (100%). No single clone could be detected when plasmids were used as the reprogramming method for PBMCs (Supplemental Table 3). Reprogramming success rates for MSCs were 100% by using both, Sendai virus or plasmids (Supplemental Table 3). The reprogramming efficiency was calculated as alkaline phosphatase-positive cell colonies from the cell number used for reprogramming. The reprogramming efficiencies of fibroblasts are in line with previous studies, indicating significantly higher rates for the Sendai virus (1.37 ± 0.4) system and the STEMCCA virus (1.30 ± 0.4) compared to the plasmid system (0.04 ± 0.01) (Figure 1(a), Table 2). It should be noted that the reprogramming efficiency was higher in fibroblasts compared to PBMCs as the parental cell source by using Sendai virus (PBMCs: 0.002 ± 0.001) (Figure 1(a)). Since plasmids resemble a cost-effective nonintegrating reprogramming method, we directly compared plasmid-based reprogramming efficiencies of FB with MSCs and PBMCs and found that human MSCs showed extremely high reprogramming efficiency (3.3 ± 1.2) by using episomal plasmids (Figure 1(b)). In contrast, reprogramming of PBMCs by plasmids was not successful (Figure 1(b)). Furthermore, we saw no significant effect of sex of the parental cells on the reprogramming results (Table 2). Importantly, we are able to generate ps-iPSCs with all three reprogramming methods from fibroblasts from donors aged 25 to 77. All generated iPSC lines showed typical hESC morphology and were positive for alkaline phosphatase (Figure 1(c)).

### 3.2. Generated iPSCs Show Characteristics of Pluripotent Cells Independent of the Reprogramming Method

**Figure 2:**
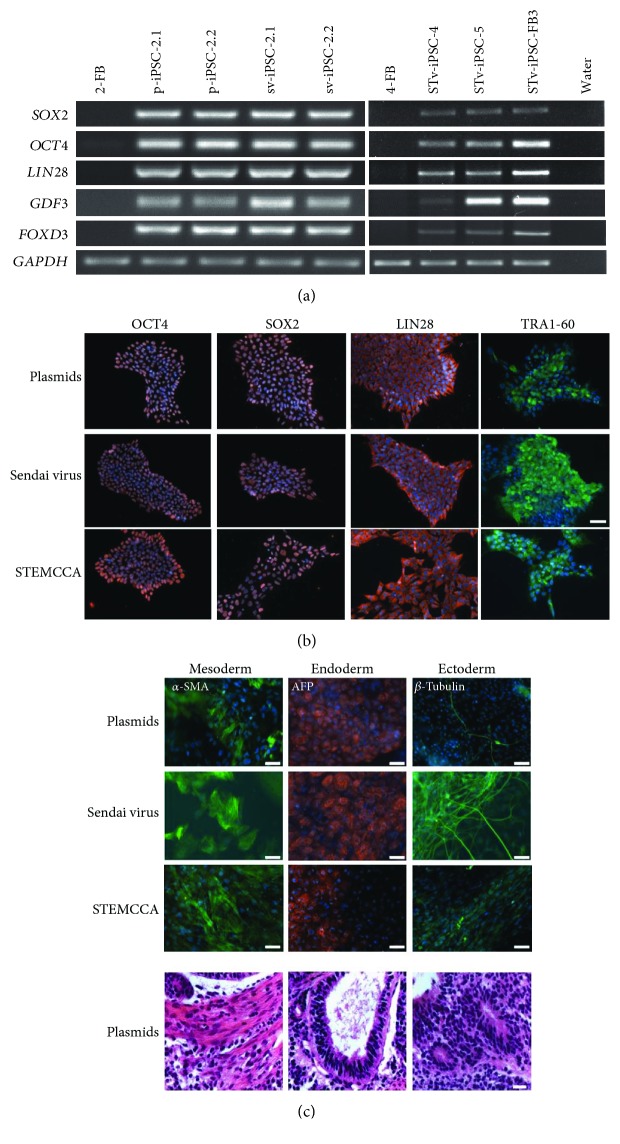
Generated iPSCs are pluripotent. (a) The iPSC lines (representative for different reprogramming methods as episomal plasmids, Sendai virus, and STEMCCA) express endogenous pluripotency marker *SOX2*, *OCT4*, *LIN28*, *GDF3*, and *FOXD3* genes at the mRNA level, which was shown by RT-PCR. iFB5 cells were used as the positive control for pluripotent iPS cells [13]. 2-FB and 4-FB are fibroblast before reprogramming. Water was used as the negative control. All other mentioned cell lines are iPSCs. (b) Expression of pluripotency markers OCT4, SOX2, LIN28, and TRA1-60 as shown by immunofluorescence staining. Scale bar: 50 *μ*m. (c) Spontaneous differentiation capacity in vitro via embryoid bodies (EBs) of three iPSC lines, representative of each reprogramming method. Immunocytochemical staining was used for analysis of differentiation capacity by protein expression of mesodermal *α*-SMA, endodermal *α*-fetoprotein (AFP), and ectodermal *β*III-tubulin exemplarily in one iPSC line generated by each reprogramming method. Scale bar: 100 *μ*m. IPSC colonies were used for teratoma formation in immunodeficient mice to test the developmental potential of iPSCs in vivo. Representative pictures of mesodermal, endodermal, and ectodermal tissues are shown from one cell line generated by the plasmid reprogramming method. Scale bar: 20 *μ*m. GAPDH was used as the loading control (a). Nuclear localization was marked with DAPI (blue) (b, c).

All iPSC lines generated for this study showed typical human pluripotent stem cell features (Figures 2(a) and 2(b)) independent of the method used for reprogramming. RT-PCR analyses displayed the activation of endogenous pluripotency genes (*SOX2*, *OCT4*, *LIN28*, *GDF3*, and *FOXD3*) in all analyzed iPSC lines compared to their parental fibroblasts (Figure 2(a)). They were positive for human pluripotent stem cell markers OCT4, SOX2, LIN28, and TRA1-60 as demonstrated with immunocytochemical staining (Figure 2(b)). Upon spontaneous differentiation via embryoid body (EB) formation, all iPSC lines differentiated into derivatives of three embryonic germ layers in vitro, as detected with immunocytochemical stainings of mesodermal alpha-smooth muscle actin (*α*-SMA), endodermal *α*-fetoprotein (AFP), and ectodermal *β*-tubulin (Figure 2(c)). The developmental potential of iPSCs in vivo was confirmed by generation of teratoma in immunodeficient mice (Figure 2(c)).

### 3.3. HIV-1 Detection by a High-Throughput PCR Method Is Usable for Footprint-Free Generated iPSCs

The retrovirus HIV-1 is able to infect human T cells and is described to be involved in the development of AIDS and adult T cell leukemia. Two RNA molecules of the retrovirus are reverse-transcribed into DNA and integrate into the host genome. A number of cell lines are known, which are latently virus-infected or actively producing viruses [18].

**Figure 3:**
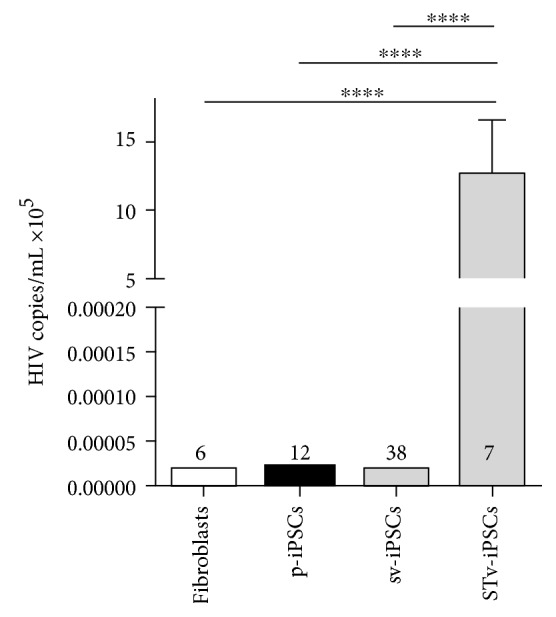
HIV-1 detection by a high-throughput PCR method in fibroblasts and iPSCs. STEMCCA-, Sendai-, and plasmid-based iPSC clones were tested for the presence of HIV-1. All STEMCCA-generated iPSC lines were positive for HIV with a titer of 12.67 × 10^5^ ± 3.9 cp/mL. Fibroblasts (FB): *n* = 6; p-iPSCs (plasmid): *n* = 12; sv-iPSCs (Sendai virus): *n* = 38; STv-iPSCs (STEMCCA): *n* = 7. *n* (numbers) are defined as HIV detection experiments of different cell types and lines generated by the mentioned reprogramming method.

We aimed to establish conditions to use the cobas® 6800/8800 system for analysis of HIV-1 contaminations of generated iPSC lines. The cobas® HIV-1 test is an in vitro nucleic acid amplification test originally developed for quantitative determination of HIV-1 in EDTA plasma of HIV-1 infected patients. The test is based on a fully automated sample preparation (extraction and purification of nucleic acids) followed by PCR amplification and detection. The sensitivity of the PCR assay was determined as about 20 copies per reaction (cp/mL). HxV H (+) C and HxV L (+) C were used as positive controls for HIV-1. As the internal negative control, human HIV-1-free plasma without HIV-1 detection was used. We tested in a total of 7 iPSC lines generated by STEMCCA virus (from 7 different donors), 38 lines generated by Sendai virus (from 26 different donors), and 12 iPSC lines generated by episomal plasmids (from 8 different donors) for the presence of HIV-1. All tested iPSC lines are already biobanked in the Translational Stem Cell group of the University Medicine Goettingen. HIV-based viruses as STEMCCA contain still fragments of the GAG and LTR regions and integrate into the host genome during the reprogramming process. Therefore, initially, we used 5 different biobanked STEMCCA-based iPSC lines as the positive control for the PCR-based cobas® 6800/8800 detection system. We were able to show that all STEMCCA-generated iPSC lines are positive for HIV with a titer of 12.67 × 10^5^ ± 1.2 cp/mL (Figure 3, Table 1). To analyze whether the infection is based on the primary material, which may be traced back to the donor, or a secondary result of personnel handling or reprogramming method, we tested the somatic fibroblasts of 3 donors (donors 3, 4, and 5; Table 1), which were used for reprogramming into STEMCCA-iPSC-3, 4, or 5. All tested fibroblasts were negative for HIV-1 (Figure 3, Table 1). Then, we tested Sendai virus and plasmid-generated iPSCs (mostly from the same patients: iPSC lines 1-7) and found that none of the nonintegrating methods for reprogramming showed detectable HIV-1 targets (Figure 3, Table 1). This data suggest that the positive HIV-1 signal in STEMCCA iPSCs is based on the integrating reprogramming method. In order to prove the utility of the high-throughput cobas® 6800/8800 system in further already biobanked patient-specific iPSC lines, we included 37 additional iPSC lines in our study (Table 3). We found again that all iPSC lines generated by nonintegrating reprogramming methods are negative for HIV detection (Table 3). Furthermore, we saw no effect of sex of the parental cells on the detection of HIV-1 in the generated iPSCs (Tables 1 and 3). In summary, our data shows that it is possible to use the high-throughput cobas® 6800/8800 system, which is normally used for detection of HIV-1 in plasma of patients, to analyze *in vitro* cell cultures such as iPSCs regarding the contamination of HIV-1.

## 4. Discussion

In the present study, we directly compared three reprogramming systems for generation of ps-iPSCs, their pluripotency capacity, and the ability to analyze HIV-1 (virus) contaminations of generated iPSC lines by using the cobas® 6800/8800 system and the cobas® HIV-1 test that was originally developed for quantitative determination of HIV-1 in EDTA plasma of HIV-1-infected patients in a high-throughput manner.

Our results show that all three reprogramming methods can be used for induction of pluripotency in somatic skin fibroblasts. The success rate and reprogramming efficiency were significantly higher by using the Sendai virus and the STEMCCA system compared to the episomal plasmids. All analyzed ps-iPSC lines are pluripotent, show phenotypical characteristics similar to already published pluripotent iPSCs, and can differentiate into all three germ layers. In accordance with the literature, we were able to show that the reprogramming efficiency is dependent on the used parental cell source. Due to the fact that the acquisition of skin biopsies is more invasive for patients than taking blood samples, we compared the reprogramming efficiencies of PBMCs as the noninvasive cell source. By using the most effective reprogramming method via Sendai virus, we detected significantly lower reprogramming efficiencies for PBMCs(0.002 ± 0.001) compared to skin fibroblasts (1.37 ± 0.4) (Figure 1(a)). Of note, reprogramming of PBMCs by plasmids was not successful. However, human mesenchymal stem cells (MSCs) used in this study were isolated from inferior nasal concha tissue from healthy individuals undergoing a conchotomy of the lower turbinate. Reprogramming of MSCs resulted in extremely high reprogramming efficiency (3.3 ± 1.2) by using episomal plasmids compared to FB and PBMC (Figure 1(b)). This comparison of low-cost reprogramming by plasmids between the 3 somatic cell types FB, PBMCs, and MSCs was never shown before and indeed confirm the major role of parental cell type in the reprogramming procedure. Our data of high reprogramming efficiencies of MSC confirms a previous study from our group, where bone marrow MSCs showed highest reprogramming efficiencies independent of the used reprogramming method [13]. Based on donors analyzed in this study, we did not obtain differences in reprogramming efficiencies dependent on sex of the parental cells (Table 2). Although, episomal plasmid reprogramming of skin fibroblasts and BPMCs into ps-iPSCs is not as effective as the STEMCCA or the Sendai virus method, episomal plasmid reprogramming still offers significant advantages over the virus systems in some reports. Plasmids can be easily isolated and purified from *E.coli* cultures, and therefore, this method is much cheaper than the commercially available viral extracts for the Yamanaka factors in the case of Sendai virus. Therefore, episomal reprogramming resembles an effective strategy to generate footprint-free iPSCs with high efficiency in MSC but lower efficiencies in FB and PBMC. Sendai virus has the advantage to be an RNA virus that does not enter the nucleus, and in addition, large amounts of proteins can be generated. This could in part explain the high reprogramming efficiency (1.37%), which is in line with a previous publication, where neonatal and adult human fibroblasts were reprogrammed with an efficiency of 1% [4]. It has to be noted that episomal and Sendai virus-based methods differ in the amount of time until losing the footprint. A somewhat lower but still high reprogramming efficiency was obtained by the use of the STEMCCA system with 1.30%. One disadvantage of this system is that after removing the 4 factors out of the genome of the iPSCs by Cre recombinase, some small virus sequences are still left.

In addition, we were able to demonstrate that the cobas® 6800/8800 system and the cobas® HIV-1 test are applicable to test in vitro generated footprint-free iPSC lines (Sendai virus or episomal plasmids), for quantitative determination of HIV-1 infections. All of our 50 already biobanked footprint-free iPSC lines showed negative HIV-1 results by the cobas® HIV-1 test. The results were confirmed by using the parental fibroblasts. In contrast, all STEMCCA-based reprogrammed iPSC lines (partly from the same parental fibroblasts as the Sendai-based iPSCs: 3-FB, 4-FB, and 5-FB are reprogrammed to sv-iPSC-3, 4, and 5 or STv-iPSC-3, 4, and 5) were used as positive controls for HIV-1, because of GAG and LTR sequences in the lentivirus-based STEMCCA vector originally established from retrovirus HIV. The Roche HIV-1 tests use the dual-target technology, including two detection probes with fluorescent reporter dyes specific against highly conserved regions of the HIV-1 genome (LTR and GAG). This explains the positive signals by the cobas® HIV-1 test in the STEMCCA virus-generated iPSCs. However, the specificity of the cobas® HIV-1 test has to be analyzed in detail in the future to consider whether this method is able to detect marginal pathogen contamination in the iPSCs. In summary, this is the first study which has demonstrated the feasibility to use a high-throughput PCR-based method (the cobas® 6800/8800 system and the PCR-based cobas® HIV-1 test) as a diagnostic tool for HIV detection to screen already banked patient-specific footprint-free iPSC lines regarding HIV-1 infection. For GMP standard procedures and in iPSC screening units, it is of exceeding importance that generated iPSC lines are virus contamination-free cultures. Since the generated iPSC lines do not carry high-risk viral contaminants, they do not represent safety risks for the personnel and could be used for drug screening experiments. This assay therefore provides a cost-effective, fast, robust, sensitive, and reliable method for routine screening.

To answer the question of which reprogramming method should be chosen for iPSCs, it has to be considered 1st whether there are long-term translational goals for the iPSCs in terms of transplantation studies for regeneration. In this case, the presence of integrated sequences in the iPSCs should be avoided, and Sendai virus or episomal plasmids should be used. If not, an infection with STEMCCA will be sufficient, as this method is usable for several cell types with high efficiency and in a cost-effective way. 2nd, it has to be clarified whether generated iPSCs need to be tested regarding virus contamination as it is common in screening units nowadays. For using the established method of the cobas® 6800/8800 system and the cobas® HIV-1 test, the STEMCCA-generated iPSCs are not usable because of false positive HIV-1 detection. Furthermore, it was shown in the past that different reprogramming methods result in transcriptomic and epigenomic differences in iPSCs [12] that need to be considered when choosing a reprogramming method. Notably, the reprograming method must not affect the differentiation potential as it has been demonstrated for the cardiac differentiation potential of iPSCs [13, 17].

## 5. Conclusion

In conclusion, we have shown that somatic cells as FB, PBMCs, and MSCs can be induced for pluripotency by nonintegrating and integrating reprogramming methods. To the best of our knowledge, we are the first who demonstrate the feasibility of the cobas® 6800/8800 system to screen patient-specific footprint-free iPSC lines regarding HIV-1 infection in a high-throughput manner. This assay, originally developed for detection of HIV-1 in plasma of HIV-1-infected patients, provides a cost-effective, fast, robust, sensitive, and reliable system for routine screening of pathogenic viruses in already biobanked footprint-free iPSC lines. In addition, our data suggests that human iPSC lines directly obtained from donors/patients not belonging to specific risk groups do not carry a high risk of HIV-1 contaminations and thus do not represent safety risks for the personnel.

## Figures and Tables

**(a):** 

Somatic cells	Cell source	Sex	HIV titer (cp/mL)
1	Skin fibroblasts from donor 1	f	<20
2	Skin fibroblasts from donor 2	f	n.a.
3	Skin fibroblasts from donor 3	m	<20
4	Skin fibroblasts from donor 4	m	<20
5	Skin fibroblasts from donor 5	f	<20
6	Skin fibroblasts from donor 6	f	n.a.
7	Skin fibroblasts from donor 7	f	<20

**(b):** 

iPSCs	Literature	Cell source	Reprogramming method	Sex	HIV titer (cp/mL)
p-iPSC-1	[19]	Skin fibroblasts	Plasmids	f	<20
sv-iPSC-1	[19]	Skin fibroblasts	Sendai virus	f	<20
p-iPSC-2	[19]	Skin fibroblasts	Plasmids	f	<20
sv-iPSC-2.1	[19]	Skin fibroblasts	Sendai virus	f	<20
sv-iPSC-2.2	[19]	Skin fibroblasts	Sendai virus	f	<20
STv-iPSC-3	Unpublished	Skin fibroblasts	STEMCCA virus	m	6.1 × 10^5^
sv-iPSC-3	Unpublished	Skin fibroblasts	Sendai virus	m	<20
STv-iPSC-4	Unpublished	Skin fibroblasts	STEMCCA virus	m	6.5 × 10^5^
sv-iPSC-4	Unpublished	Skin fibroblasts	Sendai virus	m	<20
STv-iPSC-5	Unpublished	Skin fibroblasts	STEMCCA virus	f	9.8 × 10^5^
sv-iPSC-5	Unpublished	Skin fibroblasts	Sendai virus	f	<20
p-iPSC-6.1	[19]	Skin fibroblasts	Plasmids	f	<20
p-iPSC-6.2	[19]	Skin fibroblasts	Plasmids	f	<20
p-iPSC-6.3	[19]	Skin fibroblasts	Plasmids	f	<20
p-iPSC-7.1	[19]	Skin fibroblasts	Plasmids	f	<20
p-iPSC-7.2	[19]	Skin fibroblasts	Plasmids	f	<20
sv-iPSC-7.1	[19]	Skin fibroblasts	Sendai virus	f	<20
sv-iPSC-7.2	[19]	Skin fibroblasts	Sendai virus	f	<20
STv-iPSC-FB3	[13]	Skin fibroblasts	STEMCCA virus	f	7.6 × 10^5^
STv-iPSC-FB5	[13]	Keratinocytes	STEMCCA virus	m	8.9 × 10^5^

**Table 2:** Cell lines used in this study for reprogramming efficiency.

iPSCs	Cell source	Reprogramming method	Sex	Reprogramming efficiency (%)
STv-iPSC-3	Skin fibroblasts	STEMCCA	m	1.042
p-iPSC-3	Skin fibroblasts	Plasmids	m	0.171
sv-iPSC-3	Skin fibroblasts	Sendai virus	m	3.172
STv-iPSC-4	Skin fibroblasts	STEMCCA	m	2.503
sv-iPSC-4	Skin fibroblasts	Sendai virus	m	2.916
STv-iPSC-5	Skin fibroblasts	STEMCCA	f	0.348
sv-iPSC-5	Skin fibroblasts	Sendai virus	f	1.925
p-iPSCs-8	Skin fibroblasts	Plasmids	f	0.021
sv-iPSCs-8	Skin fibroblasts	Sendai virus	f	0.122
p-iPSC-9	Skin fibroblasts	Plasmids	m	0.045
sv-iPSC-9	Skin fibroblasts	Sendai virus	m	0.169
p-iPSC-10	Skin fibroblasts	Plasmids	f	0.014
sv-iPSC-10	Skin fibroblasts	Sendai virus	f	0.649
p-iPSC-11	Skin fibroblasts	Plasmids	f	0.032
sv-iPSC-11	Skin fibroblasts	Sendai virus	f	0.680
p-iPSC-12	Skin fibroblasts	Plasmids	m	0.012
p-iPSC-13	Skin fibroblasts	Plasmids	f	0.038
p-iPSC-14	Skin fibroblasts	Plasmids	m	0.021
sv-iPSC-15	PBMCs	Sendai virus	f	0.001
sv-iPSC-16	PBMCs	Sendai virus	f	0.002
sv-iPSC-17	PBMCs	Sendai virus	f	0.003
sv-iPSCs-18	PBMCs	Sendai virus	m	0.003
p-iPSCs-19	MSCs	Plasmids	f	2.800
p-iPSCs-20	MSCs	Plasmids	m	0.37
p-iPSCs-21	MSCs	Plasmids	m	6.19
p-iPSCs-22	MSCs	Plasmids	m	3.90

**Table 3:** Additional HIV-analyzed biobanked iPSC lines.

iPSCs	Literature	Cell source	Reprogramming method	Sex	HIV titer (cp/mL)
p-iPSC-8	Unpublished	Skin fibroblasts	Plasmids	f	<20
p-iPSC-10.1	Unpublished	Skin fibroblasts	Plasmids	f	<20
p-iPSC-10.2	Unpublished	Skin fibroblasts	Plasmids	f	<20
sv-iPSC-11	Unpublished	Skin fibroblasts	Sendai virus	f	<20
sv-iPSC-15	Unpublished	PBMCs	Sendai virus	f	<20
sv-iPSC-16	Unpublished	PBMCs	Sendai virus	f	<20
sv-iPSC-18.1	Unpublished	PBMCs	Sendai virus	m	<20
sv-iPSC-18.2	Unpublished	PBMCs	Sendai virus	m	<20
sv-iPSC-23.1	Unpublished	Skin fibroblasts	Sendai virus	f	<20
sv-iPSC-23.2	Unpublished	Skin fibroblasts	Sendai virus	f	<20
sv-iPSC-24	Unpublished	Skin fibroblasts	Sendai virus	m	<20
sv-iPSC-25.1	Unpublished	Skin fibroblasts	Sendai virus	m	<20
sv-iPSC-25.2	Unpublished	Skin fibroblasts	Sendai virus	m	<20
sv-iPSC-26	Unpublished	Skin fibroblasts	Sendai virus	f	<20
sv-iPSC-27	Unpublished	Skin fibroblasts	Sendai virus	f	<20
p-iPSC-28	[19]	Skin fibroblasts	Plasmids	f	<20
sv-iPSC-29.1	Unpublished	PBMCs	Sendai virus	f	<20
sv-iPSC-29.2	Unpublished	PBMCs	Sendai virus	f	<20
sv-iPSC-30	Unpublished	Skin fibroblasts	Sendai virus	f	<20
p-iPSC-31	Unpublished	Skin fibroblasts	Plasmids	f	<20
sv-iPSC-32	Unpublished	Skin fibroblasts	Sendai virus	m	<20
sv-iPSC-33.1	Unpublished	Skin fibroblasts	Sendai virus	f	<20
sv-iPSC-33.2	Unpublished	Skin fibroblasts	Sendai virus	f	<20
sv-iPSC-34.1	Unpublished	Skin fibroblasts	Sendai virus	m	<20
sv-iPSC-34.2	Unpublished	Skin fibroblasts	Sendai virus	m	<20
sv-iPSC-35.1	Unpublished	Skin fibroblasts	Sendai virus	f	<20
sv-iPSC-35.2	Unpublished	Skin fibroblasts	Sendai virus	f	<20
sv-iPSC-36.1	Unpublished	Skin fibroblasts	Sendai virus	f	<20
sv-iPSC-36.2	Unpublished	Skin fibroblasts	Sendai virus	f	<20
sv-iPSC-37	Unpublished	Skin fibroblasts	Sendai virus	m	<20
sv-iPSC-38.1	[19]	PBMCs	Sendai virus	f	<20
sv-iPSC-38.2	[19]	PBMCs	Sendai virus	f	<20
sv-iPSC-39	Unpublished	PBMCs	Sendai virus	m	<20
sv-iPSC-40	Unpublished	PBMCs	Sendai virus	m	<20
STv-iPSC-41	[13]	Skin fibroblasts	STEMCCA virus	f	1.5 × 10^6^
STv-iPSC-42	[14]	Skin fibroblasts	STEMCCA virus	f	3.5 × 10^6^
sv-iPSC-43	[19]	Skin fibroblasts	Sendai virus	f	<20

## Data Availability

The experimental data used to support the findings of this study are included within the article or are provided as Supplementary Materials under this article.

## Abbreviations

HIV: Human immunodeficiency virus
ps-iPSC-CMs: Patient-specific induced pluripotent stem cell-derived cardiomyocytes
PBMCs: Peripheral blood mononuclear cells
FB: Fibroblasts
MSCs: Mesenchymal stem cells.

## Supplementary Materials

Supplementary Materials Supplemental Material: a high-throughput method as a diagnostic tool for HIV detection in patient-specific induced pluripotent stem cells generated by different reprogramming methods: 1. Materials and Methods: 1.1 Teratoma Formation; 1.2 Alkaline Phosphatase (ALP) Staining; 1.3 Embryoid Body (EB) Formation; 1.4 Reverse Transcriptase-Polymerase Chain Reaction (RT-PCR); 1.5 Immunocytochemical Staining; 1.6 Statistical Analysis. Supplemental Table 1: primers used in RT-PCR. Supplemental Table 2: antibodies used in immunocytochemistry. Supplemental Table 3: summary of generated ps-iPSC lines from fibroblasts and PBMCs.
