# Supplementary material for: A High-Throughput Method as a Diagnostic Tool for HIV Detection in Patient-Specific Induced Pluripotent Stem Cells Generated by Different Reprogramming Methods
Source: Stem Cells Int. 2019 Aug 5;2019:2181437. doi: 10.1155/2019/2181437 (PMC6701349; doi:10.1155/2019/2181437)
Supplement: Supplementary Materials — Supplemental Material: a high-throughput method as a diagnostic tool for HIV detection in patient-specific induced pluripotent stem cells generated by different reprogramming methods: 1. Materials and Methods: 1.1 Teratoma Formation; 1.2 Alkaline Phosphatase (ALP) Staining; 1.3 Embryoid Body (EB) Formation; 1.4 Reverse Transcriptase-Polymerase Chain Reaction (RT-PCR); 1.5 Immunocytochemical Staining; 1.6 Statistical Analysis. Supplemental Table 1: primers used in RT-PCR. Supplemental Table 2: antibodies used in immunocytochemistry. Supplemental Table 3: summary of generated ps-iPSC lines from fibroblasts and PBMCs. [file 2181437.f1.pdf]

Supplemental material:

A high throughput method as diagnostic tool for HIV detection in patient-specific induced pluripotent stem cells generated by different reprogramming methods

1. Materials and Methods:

1.1 Teratoma formation

To investigate the developmental potential of reprogrammed clones *in vivo*, hiPSCs (about 2x $1x10^6$ cells for each mouse) were injected subcutaneously into 8-week-old SCID mice as described previously [1]. Six to eight weeks after injection the mice were sacrificed and the resulting teratomas were examined after hematoxylin and eosin staining for the presence of derivatives of the three germ layers.

1.2 Alkaline Phosphatase (ALP) staining

Activity of Alkaline Phophatase (ALP) in hiPSC-colonies was detected using the ALP Staining Kit from SIGMA according to manufacturer´s protocol. Therefore hiPSC-colonies (6-cm dishes at 60% confluence) were fixed stained and analyzed by light microscopy.

1.3 Embroid body (EB) formation

For Analyzing the spontaneous differentiation capacity of the hiPSCs an embroid body (EB) formation was initialized. Therefor iPSCs were cultivated on mouse embryonic fibroblasts in hES-medium (DMEM/F12, 1% NEAA, 1% ß-mercaptoethanol, 15% knockout serum). When the hiPSCs reach a confluency of 90-100% cells were treated for 4 min with collagenase IV (200 U/mL) at 37°C and scrapped into big clusters. The clusters were gently resuspended in hES-medium with a glas pipette and transferred to an uncoated 6cm dish so that EBs formation could occure in suspension. After 24 h medium was changed to Iscove-Differentiation Medium (Iscove medium, 20% fetal calf serum, 1% NEAA, monothioglycerol (450 µmol/L)). After 8 days of cultivation, accrued EBs were collected and either used for RNA samples or for immunocytochemical staining (plated onto Geltrex coated plates and coverslips). EBs were analysed at day 8, 8+8 and 8+25.

1.4 Reverse transcriptase-polymerase chain reaction (RT-PCR)

For total RNA isolation the SV Total RNA Isolation System with on-column DNase digestion (Promega) was used. The first-strand cDNA synthesis was carried out with DNase-treated RNA (200 ng) by using Murine Leukaemia Virus Reverse Transcriptase and Oligo $d(T)_{16}$ (Applied Biosystems). One-tenth of the cDNA reaction was taken as PCR template and amplified for 25-35 cycles depending on the relative mRNA quantity with denaturation at 94 °C for 15 s, annealing at 51 °C to 66 °C for 15 s according to the primers, and elongation at 72 °C for 30 s. The forward and reverse primer sequences, the annealing temperatures and the number of cycles being performed for RT-PCR analyses were listed in Supplemental Table 1. GAPDH was used as an internal control.

1.5 Immunocytochemical staining

Cultures at different stages were fixed with 4% paraformaldehyde (PFA, Sigma) in in phosphate-buffered saline (PBS) for 20 min at room temperature and afterwards blocked with 1% BSA at 4 °C overnight. The primary antibodies were disposed for 1 h at 37 °C or overnight at 4 °C containing 0.1% Triton X-100 (Sigma) in PBS for permeabilization (when antibodies against nuclear proteins as OCT4, SOX2 and NANOG are used). The fluorescently labeled secondary antibodies showed minimal cross reactivity and were applied in 1% BSA for 1 h at 37 °C. Nuclei were count-stained with 4.6-diamino-2-phenylindole (DAPI, 0.2 ng/mL, Sigma) for 10 min at room temperature. For mounting the samples VectaShield mounting medium (VECTOR Laboratories) was used. Details regarding the appropriated antibodies are listed in Supplemental Table 2.

Images were evaluated using a fluorescence microscope (Axiovert 200, Zeiss). Detection of the FITC fluorophore was carried out by using the filter sets with excitation BP 475/40, beam splitter FT 500 and emission BP 530/50. Detection of the Cy3 fluorophore was done by using the filter sets with excitation BP 540/25, beam splitter FT 565 and emission BP 605/55. The filter sets with excitation G 365, beam splitter FT 395 and emission BP 445/50 was performed for the detection of the DAPI fluorophore.

1.6 Statistical analysis

Data were given as the mean±SEM (standard error of the mean). One way-ANOVA was used for comparison of more than two groups and multiple comparisons. A value of p less than 0.05 was considered statistically significant. *= $p \leq 0.05$, **= $p \leq 0.01$; ***= $p \leq 0.001$. Statistical

analysis was done with GraphPad prism 6 or 7. N-numbers are defined for each experiment in the figure legend.

References:

1. Guan K, Nayernia K, Maier LS, et al. Pluripotency of spermatogonial stem cells from adult mouse testis. Nature. 2006;440:1199-1203.

Supplemental Table 1: Primers used in RT-PCR

Gene name	Product size (bp)	Forward primer sequence	Reverse primer sequence	Annealing temperature	Cycle
GAPDH	258	AGA GGC AGG GAT GAT GTT CT	TCT GCT GAT GCC CCC ATG TT	55 °C	34
OCT4	218	GAC AAC AAT GAA AAT CTT CAG GAG A	TTCTGGCGCCGGTTACAGAACCA	58 °C	36
LIN28	410	AGT AAG CTG CAC ATG GAA GG	ATT GTG GCT CAA TTC TGT GC	52 °C	38
SOX2	437	ATG CAC CGC TAC GAC GTG A	CTT TTG CAC CCC TCC CAT TT	56 °C	43
GDF3	311	TTC GCT TTC TCC CAG ACC AAG GTT TC	TAC ATC CAG CAG GTT GAA GTG AAC AGC ACC	54 °C	32
FOXD3	353	GTG AAG CCG CCT TAC TCG TAC	CCG AAG CTC TGC ATC ATG AG	61 °C	38

Supplemental Table 2: Antibodies used in Immunocytochemistry

Primary antibody	Type	Dilution	Supplier
hOCT4	Polyclonal goat IgG	1:40	R&D, Minneapolis, Minnesota, USA
hSOX2	monoclonal mouse IgG2A	1:50	R&D, Minneapolis, Minnesota, USA
hLIN28	Polyclonal goat IgG	1:300	R&D, Minneapolis, Minnesota, USA

hTRA1-60	Monoclonal mouse IgM	1:200	Abcam, Cambridge, United Kingdom
hAFP	Polyclonal rabbit IgG	1:100	Dako, Hamburg, Germany
hα-SMA	Monoclonal mouse IgG2A	1:3000	Sigma Aldrich, St. Louis, Missouri, USA
β-III-tubulin	mouse IgG2A	1:1000	BioLegend, San Diego, California, USA
**Secondary antibody**	**Type**	**Dilution**	**Supplier**
Alexa Fluor 647 α-rabbit	Donkey-α-rabbit IgG	1:1000	Thermo Fisher Scientific, Waltham, Massachusetts, USA
Alexa Fluor 555 α-goat	Donkey-α-goat IgG	1:1000	Thermo Fisher Scientific, Waltham, Massachusetts, USA
Alexa Fluor 488 α-mouse	Donkey-α-mouse IgG	1:1000	Thermo Fisher Scientific, Waltham, Massachusetts, USA
Alexa Fluor 488 α-mouse	Goat-α-mouse IgG+IgM	1:500	Thermo Fisher Scientific, Waltham, Massachusetts, USA
Cyanine Cy3 α-mouse	Goat-α-mouse IgG+IgM	1:300	Jackson Immuno Research, West Grove, Pennsylvania, USA

Supplemental Table 3 Summary of generated ps-iPSC lines from fibroblasts and PBMCs

Cell sources	Reprogramming system	Number of donors analyzed	Number of donors with iPSCs generated
**Fibroblasts (FB)**	plasmids	18	16 (89%)
	Sendai virus	25	25 (100%)
	STEMCCA	5	5 (100%)
**PBMCs**	plasmids	5	0 (0%)
	Sendai virus	6	6 (100%)
	STEMCCA	n. a.	n. a.
**MSCs**	plasmids	4	4 (100%)
	Sendai virus	4	4 (100%)
	STEMCCA	n. a.	n. a.
